# Arthritis prevalence is associated with metabolic syndrome risk factors but not with physical activity in middle-aged and older patients - a cross-sectional study

**DOI:** 10.1186/s12877-024-04859-9

**Published:** 2024-03-08

**Authors:** Fanji Qiu, Jinfeng Li, Liaoyan Gan, Kirsten Legerlotz

**Affiliations:** 1 Movement Biomechanics, Institute of Sport Sciences, Humboldt-Universität zu Berlin, Unter den Linden 6, 10099 Berlin, Germany; 2 Department of Kinesiology, Iowa State University, 50011 Ames, USA IA; 3 Alberta International School of Recreation, Sport and Tourism of Beijing Sport University, Beijing Sport University, 572423 Lingshui, China; 4 Faculty of Kinesiology, Sport, and Recreation, University of Alberta, T6G2R3 Edmonton, Canada

**Keywords:** Arthritis, Rheumatism, Prevalence, Middle-aged and older, Physical activity

## Abstract

**Background:**

In light of the aging population, increasingly suffering from the metabolic syndrome (MS), strategies need to be developed to address global public health challenges known to be associated with MS such as arthritis. As physical activity (PA) may play a crucial role in tackling those challenges, this study aimed to determine the association between the number of MS risk factors, PA and arthritis in people ≥ 50 years old.

**Methods:**

Data from the Survey of Health, Ageing, and Retirement in Europe (SHARE) were used to estimate the prevalence of arthritis and MS risk factors in the European population ≥ 50 years and to evaluate the associations between MS risk factors, PA and arthritis. Binary logistic regression was performed to calculate the odds ratio of different factors.

**Results:**

73,125 participants were included in the analysis. 55.75% of patients stated at least one of the three MS risk factors. The prevalence of rheumatoid arthritis (RA) and osteoarthritis (OA)/other rheumatism among ≥ 50 years population was 10.19% and 19.32% respectively. Females showed a higher prevalence of arthritis than males. Prevalence did not differ between groups with different levels of PA. Arthritis prevalence was positively correlated with the number of MS risk factors (*P* < 0.01) but not with PA (*P* > 0.05).

**Conclusion:**

Middle-aged and older Europeans with multiple comorbidities suffered from RA, OA or other rheumatism more frequently than participants with fewer comorbidities, while the level of physical activity was not associated with the number of metabolic risk factors in patients with RA and OA/other rheumatism.

**Supplementary Information:**

The online version contains supplementary material available at 10.1186/s12877-024-04859-9.

## Background

Rheumatoid arthritis (RA) and Osteoarthritis (OA) are common joint diseases with slow disease progression and high disability rates [1, 2]. While both diseases affect joint function, the anatomical location of the predominantly affected joints as well as the pathogenesis differ. RA is an autoimmune disease characterized by persistent joint inflammation. It typically affects small joints such as the hand and can lead to deformity [3, 4]. The global prevalence of RA was 0.46% [5], while some European countries have reported prevalence rates of RA ranging from 0.31 to 0.67% [6, 7]. OA is a degenerative joint disease which leads to cartilage erosion, osteophyte formation, and synovial inflammation and is marked by pain, joint stiffness and impaired physical function [1, 8, 9]. OA mainly occurs in the larger joints of the knee, hip, and hand [8, 9]. The prevalence of OA increases with age, and in European countries, the prevalence rate is 30.4% [10]. Both RA and OA result in a huge burden on socioeconomic expenditures of public health systems [11] and have become major global public health challenges [12].

In recent years, evidence has indicated that the metabolic syndrome (MS) plays a role in the development of arthritis [13, 14], with diabetes mellitus (DM), hypertension (HTN) and hypercholesterolemia (HC) being identified as risk factors for RA and OA [14–17]. DM is associated with a dose-dependent risk for OA, as for each unit increase in blood glucose, the risk of developing knee OA increases by 1.7% [18]. The prevalence of DM worldwide was 8.3% [19], while the prevalence of DM increases to 12% among RA patients [16]. Elevated total cholesterol levels have also been linked to an increased risk of RA in females [20]. Thus, with an increase in the incidence of metabolic disease one may also see an increase in the incidence of arthritis.

Strategies must thus be developed to tackle the global public health challenge of both arthritis and metabolic diseases. In this context, physical activity (PA) could play an important role as a counter measure. The WHO recommends that adults aged 18–65 engage in at least 150 min of moderate-intensity PA per week, and suggests that even individuals with chronic diseases can benefit from PA, as the advantages associated with PA typically outweigh the risks [21]. For conditions like RA, OA, and other rheumatism-related ailments, regular PA has proven to be an effective treatment and management tool [22]. In older patients with RA, both aerobic and resistance exercises have induced positive effects on aerobic capacity, endurance, and strength-related physical fitness [23]. For those with OA, moderate exercise can increase glycosaminoglycans (GAGs) in the cartilage of individuals at high risk of OA [24]. Furthermore, physical activity has been shown to significantly reduce blood pressure, blood glucose and cholesterol levels [25]. However, it is worth noting that the relationship between PA and arthritis prevalence is not fully understood, as there are conflicting findings in different studies [26]. Therefore, further research is needed to gain a comprehensive understanding of the impact of PA on arthritis prevalence.

Despite the wealth of research on individual factors, limited attention has been given to exploring the combined effect of multiple MS risk factors on the prevalence of arthritis. Further investigations into the impact of the number of MS risk factors on arthritis prevalence are warranted to gain a more comprehensive understanding of the relationship between these conditions.

The prevalence of RA and OA increases with age [27, 28], with a prevalence of 24.7-31.4% in individuals over 50 years of age [29, 30]. This has raised further concerns, as over 40 million people in Europe are already affected by arthritis [31] and the population aged 65 and above is projected to reach 129.8 million in the EU by 2050 [32]. Therefore, understanding the associations between the number of MS risk factors, PA and arthritis in the population ≥ 50 years of age is essential to understand disease trends and to develop effective prevention strategies.

The objective of this study was to examine the association between the number of MS risk factors, PA and arthritis. We hypothesized that: (1) the likelihood that a patient has arthritis increases as the number of MS risk factors increases, and (2) higher levels of PA are associated with lower arthritis prevalence.

## Methods

### Study population and study design

For our analysis, data from the Survey of Health, Ageing, and Retirement in Europe (SHARE) Wave 7 were used. SHARE is a longitudinal population study database that includes multidisciplinary information on health, social networks, and the socio-economic status of approximately 140,000 adults aged 50 years and above in Europe (27 European countries and Israel) [33–35]. SHARE has collected data every two years since 2004, for a total of eight waves of data collection. Wave 7 began in March 2017 and ended in October 2017. Approximately 80,000 interviews, including end-of-life interviews, were conducted during Wave 7 using computer-assisted telephone interviewing (CAPI). The relevant ethics committees of all participating countries approved SHARE, and all participants provided written informed consent [36].

**Fig. 1:**
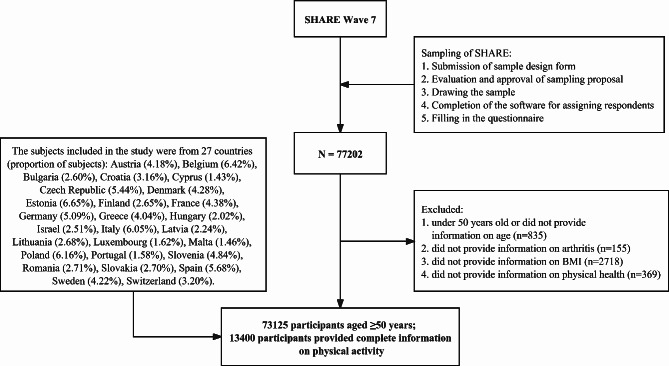
Flow diagram of participant recruitment [35]

This study applied a cross-sectional design and assessed all dependent and independent variables at the same time point. Our final data set included 73,125 community residents aged ≥ 50 years (Fig. 1). All included subjects provided information on OA/ other rheumatism and RA. A total of 13,400 participants provided complete information on PA. Incomplete data sets were excluded to avoid potential bias caused by missing data.

### Demographic and health associated information

Demographic (age, body mass index (BMI), BMI category, country and sex) and health associated characteristics (DM/HBS: Diabetes or high blood sugar; HBC: High blood cholesterol; HBP/HTN: High blood pressure or hypertension; PA: physical activity) of the interviewed persons were analyzed. BMI categories were defined as follows: underweight (below 18.5 kg/m^2^ ), normal (18.5–24.9 kg/m^2^), overweight (25-29.9 kg/m^2^) and obese (30 kg/m^2^ and above) [37].

### Physical activity

The assessment of PA level was based on two questions: (1) “How often do you engage in vigorous physical activity, such as sports, heavy housework, or a job that involves physical labor?” (2) “How often do you engage in activities that require a moderate level of energy such as gardening, cleaning the car, or doing a walk?” Participants answered those questions by choosing one of four different options: 1. More than once a week; 2. Once a week; (3) One to three times a month; (4) Hardly ever, or never.

### Metabolic diseases and risk factors for metabolic syndrome

Information on the prevalence of RA, OA/other rheumatism, DM/HBS, HBP/HTN and HBC were collected through a questionnaire about physical health answered by the participants. The participants were asked: ’’Has a doctor ever told you that you had/ Do you currently have any of the conditions on this card? [With this we mean that a doctor has told you that you have this condition, and that you are either currently being treated for or bothered by this condition.]”. Participants answered those questions by choosing one of two different options: (1) Selected; (2) Not selected. The questionnaire had listed some of the conditions individually (such as “rheumatoid arthritis” and “high blood cholesterol”) while others were listed in combination (such as “high blood pressure or hypertension”; “diabetes or high blood sugar” and “osteoarthritis or other rheumatism”). Conditions that were listed in combination, such as OA and other rheumatism, are thus counted together. The number of MS risk factors was calculated for each participant.

### Covariates

Variables that could have had an impact on RA and OA/other rheumatism were considered. Key variables were: (1) PA, including two intensities (vigorous and moderate); (2) demographic variables, including age, BMI, country (sorted by country ID), and sex (male, female).

### Statistical analysis

All analyses were performed using SPSS software (version 26, IBM: Armonk, NY, USA). When considering an OA prevalence of 30.4% [10] and an RA prevalence of 2.5% [38], with a 95% confidence interval and 95% power and calculated with Cochran’s sample size formula [39], the minimum sample size required is 325. Descriptive analyses described baseline characteristics of the study population: (1) age, (2) BMI, (3) BMI category, (4) country, and (5) sex. Means with standard deviation (SD) were calculated. We also calculated the prevalence of DM/HBS, HBC, HBP/HTN, RA and OA/other rheumatism in the study population according to the number of MS risk factors. Chi-square test or Fisher’s exact test were used for categorical variables to determine associations. Univariate binary logistic regression or multivariate binary logistic regression were used to evaluate the cross-sectional associations between independent variables (number of MS risk factors), covariates and dependent variables (RA and OA/other rheumatism). Odds ratios (OR), 95% confidence intervals, and *P*-values were calculated. We considered *P*-values < 0.05 to be statistically significant. The categories set as reference categories were: 0 MS risk factor, and hardly ever or never conduct vigorous/ moderate PA. The analysis was conducted in two steps: firstly, in the crude analysis, the independent variables were put into the model with each of the two types of arthritis; then, in the adjusted analysis, all variables were included in one model for analysis.

Multicollinearity analysis was performed to detect collinearity in the applied regression models. Collinearity was considered to be strong when the maximum variance inflation factor (VIF) value was greater than 5 [40] and the tolerance value was less than 0.20 [41].

## Results

### Descriptive subject characteristics

From all participants aged 50 years or older (*n* = 76,367), after excluding those with missing information on physical health, 73,125 participants were included in the analysis, with females accounting for 56.34%. The mean age of all participants was 68.49 ± 9.66 years (Table 1). 55.75% of all participants reported at least one MS risk factor, which was a significantly larger number compared to those who stated not to suffer from any MS risk factors (*P* < 0.05).

**Table 1:** The Characteristics of patients by number of MS risk Factors

	No. of MS risk factors				
	0	1	2	3	All
**N (%)**	32 357 (44.25)	24 255 (33.17)	12 978 (17.75)	3 535 (4.83)	73 125
**Age [y]**	66.31 ± 9.68	69.64 ± 9.52	70.98 ± 8.95	71.47 ± 8.52	68.49 ± 9.66
**Female [%]**	55.87	57.12	56.54	54.57	56.34
**MS risk factors [%]**					
HBP/HTN	0	69.41	93.16	100	44.39
HBC	0	21.43	73.87	100	25.05
DM/HBS	0	9.16	32.97	100	13.72
**Arthritis [%]**					
RA	6.84^a^	11.01^b^	14.55^c^	19.15^d^	10.19
OA/other rheumatism	15.17^a^	20.84^b^	24.53^c^	27.84^d^	19.32
**BMI [kg/m²]**	26.03 ± 4.22	27.53 ± 4.67	27.43 ± 7.37	30.01 ± 5.34	27.17 ± 4.69
**BMI by category [%]**					
Underweight (< 18.5) (*n* = 877)	1.74^a^	0.96^b^	0.54^c^	0.31^c^	1.20
Normal (18.5-<25) (*n* = 24 365)	42.24^a^	29.66^b^	22.60^c^	16.15^d^	33.32
Overweight (25-<30) (*n* = 30 471)	40.18^a^	43.48^b^	42.93^b^	38.27^a^	41.67
Obese (30-<40) (*n* = 17 412)	15.84^a^	25.90^b^	33.93^c^	45.26^d^	23.81

a, b, c, d: significant differences were found between the prevalence of different numbers of MS risk factors, with significant differences between different letters and no significant differences for the same letter. Age and BMI are presented as mean ± SD.

Abbreviations: BMI: body mass index; HBS: high blood sugar; DM: diabetes mellitus; HBC: High blood cholesterol; HBP: High blood pressure; HTN: hypertension; IQR, interquartile range; MS: metabolic syndrome; OA: osteoarthritis; RA: rheumatoid arthritis; SD: standard deviation; y: year

10.19% (*n* = 7448) of all participants were diagnosed/currently having RA, 19.32% (*n* = 14,129) were diagnosed/currently having OA/other rheumatism, 44.39% (*n* = 32,460) were diagnosed/ currently having HBP/HTN, 25.05% (*n* = 18,321) were diagnosed/currently having HBC, and 13.72% (*n* = 10,035) were diagnosed/currently having DM/HBC. The prevalence of OA/other rheumatism and RA increased significantly with the increase in the number of MS risk factors (*P* < 0.001) (Table 1).

Of those participants who reported engagement in sports or activities that were vigorous, 27.43% (*n* = 3675) exercised more than once a week, 14.29% (*n* = 1915) exercised once a week and 11.00% (*n* = 1474) exercised one to three times a month, while 47.28% (*n* = 6336) hardly ever/never exercised. Of all participants who reported engagement in moderate activities 65.46% (*n* = 8771) exercised more than once a week, 14.00% (*n* = 1876) exercised once a week, and 5.65% (*n* = 757) exercised one to three times a month, while 14.90% (*n* = 1996) hardly ever/never exercised. The prevalence of RA and OA/other rheumatism did not differ between groups engaging in different levels of PA (Table 2).

**Table 2:** The Characteristics of patients by level of PA

	Hardly ever, or never		1–3 times a month		Once a week		More than once a week	
	Vigorous PA	Moderate PA	Vigorous PA	Moderate PA	Vigorous PA	Moderate PA	Vigorous PA	Moderate PA
**N (%)**	6336 (47.28)	1996 (14.90)	1474 (11.00)	757 (5.65)	1915 (14.29)	1876 (14.00)	3675 (27.43)	8771 (65.46)
**Age [y]**	69.28 ± 9.62	69.35 ± 9.61	69.29 ± 9.61	69.39 ± 9.92	69.12 ± 9.32	69.00 ± 9.55	69.12 ± 9.57	69.21 ± 9.52
**Female[%]**	56.38	56.36	56.38	59.71	55.04	55.49	55.73	55.72
**No. of MS risk factors [%]**								
0	42.77	43.89	41.72	42.8	43.45	40.83	44.68	43.81
1	32.62	32.77	35.14	32.5	33.89	34.33	32.68	32.96
2	19.32	18.99	18.39	19.55	17.6	19.67	17.71	18.09
3	5.29	4.86	4.75	5.15	5.07	5.17	4.93	5.13
**Prevalence of Arthritis [%]**								
RA	9.36	9.67	10.18	9.77	8.93	9.75	9.74	9.37
OA/other rheumatism	22.65	22.39	20.62	19.68	20.47	21.48	23.16	22.61

Abbreviations: PA: physical activity. Age is presented as mean ± SD.

### Results of logistic regression

According to the crude analysis (Tables 3 and 4), one and more MS risk factors were positively associated with OA/other rheumatism and RA (*P* < 0.01), compared to no MS risk factors. The multicollinearity analysis did not detect signs of redundancy amongst variables (VIF < 5, Tolerance > 0.1).

**Table 3:** Association between number of MS risk factors and OA/other rheumatism and RA

	OA/other rheumatism		RA	
	Unadjusted OR	Adjusted OR	Unadjusted OR	Adjusted OR
0	Reference	Reference	Reference	Reference
1	1.472 (1.410–1.538) *	1.295 (1.237–1.354) *	1.685 (1.588–1.787) *	1.412 (1.329–1.501) *
2	1.819 (1.730–1.912) *	1.508 (1.430–1.590) *	2.319 (2.173–2.475) *	1.810 (1.691–1.937) *
3	2.158 (1.993–2.337) *	1.715 (1.577–1.864) *	3.227 (2.936–3.545) *	2.382 (2.158–2.628) *

Numbers presented as OR (corresponding 95% CI).

Unadjusted OR: obtained by putting the number of MS risk factors in the model

Adjusted OR: obtained by putting the number of MS risk factors in the model as well as the covariates (age, sex, BMI, and country). * = *P* < 0.01

**Table 4:** Association between number of MS risk factors and OA/other rheumatism and RA in subjects who provided information on PA

	OA/other rheumatism		RA	
	Unadjusted OR	Adjusted OR	Unadjusted OR	Adjusted OR
0	Reference	Reference	Reference	Reference
1	1.361 (1.237–1.499) *	1.221 (1.105–1.349) *	1.575 (1.365–1.817) *	1.375 (1.188–1.593) *
2	1.680 (1.505–1.877) *	1.395 (1.241–1.568) *	2.270 (1.945–2.650) *	1.815 (1.544–2.133) *
3	1.858 (1.556–2.219) *	1.422 (1.179–1.715) *	2.738 (2.177–3.442) *	2.034 (1.599–2.587) *

Numbers presented as OR (corresponding 95% CI).

Unadjusted OR: obtained by putting the number of MS risk factor in the model

Adjusted OR: obtained by putting the number of MS risk factors in the model as well as the covariates (PA, age, sex, BMI, and country). * = *P* < 0.001

For all subjects, when age, BMI, country, and sex were included in the model (Table 3), subjects with one MS risk factor, two MS risk factors, and three MS risk factors positively associated with RA and OA/other rheumatism prevalence compared to no MS risk factor (*P* < 0.001). For subjects who provided PA information (Table 4), the regression model added two exercise intensities besides demographic variables, and MS risk factors remained to be positively associated with RA and OA/other rheumatism prevalence (*P* < 0.001).

We also explored the association between covariates and outcomes (Table 5). The results of the regression model that included all subjects showed that higher age, higher BMI, and female sex were positively associated with OA/other rheumatism and RA prevalence (*P* < 0.01). The model that included subjects who provided information on PA revealed that higher age, higher BMI, and female sex were positively associated with OA/other rheumatism prevalence (*P* < 0.01). However, there was no association between PA and OA/other rheumatism and RA prevalence (*P* > 0.05), while this result was not affected by sex.

**Table 5:** Association between covariates and OA/other rheumatism and RA

	OA/other rheumatism		RA	
	All	With PA information	All	With PA information
Age	1.026 (1.024–1.028) *	1.028 (1.023–1.032) *	1.034 (1.032–1.037) *	1.037 (1.031–1.044) *
Sex^#^	2.070 (1.989–2.154) *	2.168 (1.985–2.367) *	1.944 (1.845–2.049) *	2.040 (1.797–2.315) *
BMI	1.035 (1.031–1.039) *	1.037 (1.027–1.046) *	1.041 (1.036–1.047) *	1.035 (1.022–1.048) *
**Vigorous PA**	-	0.996 (0.961–1.032)	-	0.971 (0.923–1.021)
Hardly ever, or never	-	Reference	-	Reference
1–3 times a month	-	0.878 (0.758–1.017)	-	1.120 (0.918–1.366)
Once a week	-	0.881 (0.771–1.008)	-	1.001 (0.828–1.210)
More than once a week	-	1.028 (0.923–1.146)	-	1.114 (0.954-1.300)
**Moderate PA**	-	0.980 (0.940–1.022)	-	1.023 (0.964–1.084)
Hardly ever, or never	-	Reference	-	Reference
1–3 times a month	-	0.841 (0.678–1.042)	-	0.951 (0.710–1.273)
Once a week	-	0.985 (0.837–1.159)	-	0.979 (0.780–1.228)
More than once a week	-	1.041 (0.913–1.187)	-	0.929 (0.772–1.118)

Numbers presented as OR (corresponding 95% CI).

OR: (1) for all subjects, obtained by putting the number of MS risk factors in the model as well as covariates (age, sex, BMI, and country); (2) for subjects with PA information, obtained by putting the number of MS risk factors in the model as well as covariates (PA, age, sex, BMI, and country). * = *P* < 0.01

#: male as reference

## Discussion

Our analysis revealed that the number of MS risk factors was positively associated with the prevalence of OA/other rheumatism and RA in the population aged ≥ 50 years in the largest yet investigated sample size. The results of the presented study are consistent with previous research, showing that the prevalence of both RA and OA/other rheumatism is higher in females than in males across different age groups [42]. We observed no correlation between PA and arthritis prevalence, regardless of sex.

Middle-aged and older Europeans with multiple comorbidities suffered from RA, OA or other rheumatism more frequently than those with fewer comorbidities while the level of physical activity was not related to the number of metabolic risk factors in patients with RA and OA/other rheumatism. It needs to be highlighted though, that the cross-sectional design of our analysis does not allow to derive any cause-and-effect relationships from this observation. Thus, it remains unclear if the metabolic changes associated with the metabolic syndrome have contributed to development or progression of any form of rheumatism, as suffering from RA, OA or other rheumatism may equally have led to changes in lifestyle which eventually resulted in symptoms of MS. In addition, the persistent inflammation in patients with chronic inflammatory rheumatic conditions may affect other organ systems. This is supported by a higher 3-year incidence of cardiovascular disease detected in RA patients compared to controls [43, 44]. Furthermore, adverse effects associated with medical treatment as well as pain medication, which is known to be prescribed to patients with chronic inflammatory rheumatic conditions more frequently than to controls [43], may have contributed to the high prevalence of comorbidities.

However, it is alarming that over half of the studied population presented with at least one risk factor for metabolic syndrome. The results of our study, conducted with a substantial sample size, underlined the critical importance of early identification and strict management of MS risk factors. Hypertension (HBP) has been associated with an increased risk of vascular disease and plays a role in the development of osteoarthritis (OA) by intermittently reducing blood flow through small blood vessels in the subchondral bone [45, 46]. Diabetes has been linked to higher oxidative stress and pro-inflammatory cytokines, negatively affecting joints by accumulating advanced glycation end products in tissues exposed to chronically high glucose concentrations [46]. Dyslipidemia leads to excessive lipid deposition in osteoarthritic chondrocytes, disrupting the balance between fat formation and cartilage formation [47]. Moreover, these metabolic diseases, including HBP, HBC, diabetes, and RA, can interact with each other, further increasing the risk of cardiovascular disease [48]. In light of these findings, it is crucial for healthcare professionals to proactively address and manage MS risk factors to mitigate their influence on arthritis prevalence and associated complications. Early intervention and effective control of MS risk factors can play a pivotal role in enhancing overall health outcomes and quality of life for individuals with arthritis and related conditions.

Contrary to our hypothesis, there was no association between PA and arthritis prevalence, nor was it identified as protective factor against arthritis, neither in men nor in women. A cross-sectional study involving 1415 participants with an average age of 73 years observed no association between habitual physical activity and knee OA, while PA was found to increase the formation of osteophytes [49]. Another study focusing on individuals aged 63–91 years revealed that heavy physical activity posed a significant risk factor for knee OA, particularly in older and obese populations [50]. Thus, potential metabolic benefits of PA for the older population may be offset by the negative side-effects of mechanical loading. Nevertheless, the latest consensus statement emphasizes the importance of increasing PA for individual health [51]. Furthermore, physical exercise has been shown to relieve pain and improve functionality across different types of arthritis [22], to reduce cartilage loss in patients with OA [24] and to improve aerobic capacity, endurance, and strength in patients with RA [23]. Compared to weightbearing activities such as weight lifting and long-distance running, which can increase the prevalence of OA [52], swimming may be more beneficial for joint health due to reduced joint compression during movement [53]. Therefore, we still recommend that middle-aged and older subjects, including patients with arthritis, engage in regular PA, while it needs to be ensured that patients exercise under the guidance of physical activity specialists, who control that appropriate levels of mechanical loading are applied.

Increased age is associated with increased prevalence of OA/other rheumatism and RA. Due to the aging of the general population the number of affected patients may thus increase in the future. This is in line with the results of other studies based on health care data and health surveys conducted in Europe [27, 28]. A Swedish study analyzing more than 500,000 people aged ≥ 45 years has calculated that nearly 30% of the population will require OA-related medical consultations by 2032 [54], and combined with our findings, it is predictable that the burden on the healthcare system will progressively increase as the aging progresses. National population-based studies conducted in the UK and France reported RA prevalence rates of 0.31–0.67% [6, 7]. That our study detected a much higher RA prevalence rate of 10.29% may result from the higher age of the investigated subjects, as the above-mentioned studies included all adults aged ≥ 18. Regarding the prevalence of OA, it is essential to note that radiographically defined OA is determined based on radiographs, while the diagnosis of symptomatic OA requires radiographic evidence and the presence of regular joint pain. Therefore, even an investigation conducted within the same population may detect differing prevalence rates for knee OA, with higher rates for radiographically defined (27.8%) than for symptomatic knee OA (16.7%) [55].

An increased BMI is associated with increased prevalence of OA/other rheumatism and RA. In middle-aged to elderly subjects with an average age of 56, obesity was associated with a 58% increase in the risk of OA [56]. In addition, a high BMI exerts a major causal effect on the risk of OA at weight-bearing joints [57]. It is most likely and predominantly the increased mechanical stress acting on weight-bearing joints which contributes to the development or progression of OA, as no causal association has been detected between BMI and OA of non-weight bearing joints of the hand [57, 58]. Regarding RA pathogenesis, obesity may promote inflammation, thereby modestly increasing the risk to develop RA [59]. A cohort study on subjects with an average age of 53.74 years found that individuals who successfully reduced their weight from obesity to non-obesity may reduce the increased risk of arthritis associated with obesity. This may be attributed to both a reduction in systemic inflammation and a reduction in mechanical stress acting on joints [60]. In our study population, 65.48% were classified as overweight, which highlights the importance to reduce obesity in middle-aged and older populations in order to foster joint health and function.

### Limitations

First, the cross-sectional design does not allow to demonstrate causal relationships between MS risk factors, PA and arthritis, nor does it allow to determine the effect of prevention or treatment of MS risk factors on the risk of RA and OA/other rheumatism. We did not have access to all possible MS risk factors for analysis, such as family history of metabolic disease, nor did we have information on important lifestyle habits (e.g., alcohol consumption, diet, and smoking) that could potentially affect the results. Another limitation is that although SHARE uses a standardized retrospective self-reporting procedure for data collection, recall bias may still lead to underreporting. Meanwhile, within large-scale multinational studies, national differences in the diagnosis of specific diseases e.g. resulting from differences in the health care system or medical education may have led to possible bias. Finally, it is important to note that the prevalence of OA/other rheumatism in this study was calculated by combining multiple diseases, so the prevalence of OA/other rheumatism needs to be interpreted with caution.

## Conclusion

In conclusion, current research indicates that among individuals aged ≥ 50 years, the more comorbidities a patient has, the higher the likelihood that a patient has also OA/ other rheumatism and RA. The level of physical activity is thereby neither related to the number of metabolic risk factors in patients with RA and OA/ other rheumatism nor does it lower arthritis prevalence. The higher prevalence in females also emphasize the importance of paying attention to sex differences in the investigation of the etiology of arthritic diseases.

### Electronic supplementary material

Below is the link to the electronic supplementary material.

Supplementary Material 1

## Data Availability

SHARE data is available through individual user registration. All information about the application and registration process can be found at https://share-eric.eu/data/become-a-user (accessed on 22 Feb 2023).

## Abbreviations

BMI: Body mass index
DM: Diabetes mellitus
GAGs: Glycosaminoglycans
HBC: High blood cholesterol
HBP: High blood pressure
HBS: High blood sugar
HC: Hypercholesterolemia
HTN: Hypertension
IQR: Interquartile range
MS: Metabolic syndrome
OA: Osteoarthritis
OR: Odds ratios
PA: Physical activity
RA: Rheumatoid arthritis
SD: Standard deviation
VIF: Variance inflation factor
y: Year
