# Supplementary material for: Arthritis prevalence is associated with metabolic syndrome risk factors but not with physical activity in middle-aged and older patients - a cross-sectional study
Source: BMC Geriatr. 2024 Mar 8;24:242. doi: 10.1186/s12877-024-04859-9 (PMC10924363; doi:10.1186/s12877-024-04859-9)
Supplement: Supplementary file 1 — Supplementary Material 1 [file 12877_2024_4859_MOESM1_ESM.docx]

Country ID:

11 = Austria, 12= Germany, 13 = Sweden, 15 = Spain, 16 = Italy , 17 = France, 18 = Denmark, 19 = Greece, 20 = Switzerland, 23 = Belgium, 25 = Israel, 28 = Czech Republic, 29 = Poland, 31 = Luxembourg, 32 = Hungary, 33 = Portugal, 34 = Slovenia, 35 = Estonia, 47 = Croatia, 48 = Lithuania, 51 = Bulgaria, 53 = Cyprus, 55 = Finland, 57 = Latvia, 59 = Malta, 61 = Romania, 63 = Slovakia

Table. The Characteristics of patients by number of MS risk Factors (with PA information)

|  | No. of MS risk factors | | | | |
| --- | --- | --- | --- | --- | --- |
|  | 0 | 1 | 2 | 3 | All |
| **N [%]** | 5799 (43.28) | 4435 (33.10) | 2483 (18.53) | 683 (5.10) | 13400 |
| **Age, mean (SD), y** | 67.15 (9.72) | 69.91 (9.29) | 71.95 (8.84) | 72.29 (8.49) | 69.21 (9.56) |
| **Median (IQR), y** | 66.00 (60.00-73.00) | 69.00 (63.00-76.00) | 71.00 (65.00-78.00) | 72.00 (66.00-79.00) | 68.00 (62.00-76.00) |
| **Female, %** | 57.01 | 55.47 | 54.81 | 55.94 | 55.34 |
| **MS risk factors [%]** |  |  |  |  |  |
| HBP or HTN | 0 | 65.30 | 91.74 | 100 | 43.71 |
| HBC | 0 | 26.29 | 77.69 | 100 | 28.19 |
| DM or HBS | 0 | 8.41 | 30.57 | 100 | 13.54 |
| **Arthritis [%]** |  |  |  |  |  |
| RA | 6.55^a^ | 9.94^b^ | 13.73^c^ | 16.11^c^ | 9.49 |
| OA/other rheumatism | 18.33^a^ | 23.40^b^ | 27.39^c^ | 29.43^c^ | 22.25 |
| **BMI, mean (SD)** | 25.76 (4.13) | 26.24 (6.73) | 27.01 (6.97) | 29.70 (5.12) | 26.87 (4.48) |
| **BMI by category [%]** |  |  |  |  |  |
| Underweight (<8.5)  (n = 167) | 1.85^a^ | 1.01^b^ | 0.56^b^ | 0.15^b^ | 1.25 |
| Normal (18.5-<25) (n = 4 641) | 44.37^a^ | 30.30^b^ | 24.41^c^ | 17.28^d^ | 34.63 |
| Overweight (25-<30) (n = 5 740) | 39.39^a^ | 45.70^b^ | 46.15^b^ | 41.43^b^ | 42.84 |
| Obese (30-<40) (n = 2 852) | 14.40^a^ | 22.98^b^ | 28.88^c^ | 41.14^d^ | 21.28 |
| **PA [%]** |  |  |  |  |  |
| Vigorous PA |  |  |  |  |  |
| Hardly ever, or never | 47.02 | 46.84 | 49.36 | 49.65 | 47.28 |
| 1-3 times a month | 10.55 | 11.45 | 10.72 | 10.49 | 11.00 |
| Once a week | 14.16 | 14.57 | 13.55 | 14.13 | 14.29 |
| More than once a week | 28.27 | 27.15 | 26.37 | 25.73 | 27.43 |
| Moderate PA |  |  |  |  |  |
| Hardly ever, or never | 15.13 | 15.04 | 15.26 | 14.55 | 14.90 |
| 1-3 times a month | 5.57 | 5.57 | 5.94 | 5.59 | 5.65 |
| Once a week | 13.14 | 14.44 | 14.76 | 14.27 | 14.00 |
| More than once a week | 66.17 | 64.95 | 64.04 | 65.59 | 65.46 |

Abbreviations: PA: physical activity.
